# Alisol A 24-acetate protects against brain microvascular endothelial cells injury through inhibiting miR-92a-3p/tight junctions axis

**DOI:** 10.18632/aging.203094

**Published:** 2021-06-04

**Authors:** Lu Lu, Taotao Lu, Julian Shen, Xinru Lv, Wei Wei, Hong Wang, Xiehua Xue

**Affiliations:** 1 The Affiliated Rehabilitation Hospital, Fujian University of Traditional Chinese Medicine, Fuzhou 350003, China; 2 College of Rehabilitation Medicine, Fujian University of Traditional Chinese Medicine, Fuzhou 350112, China

**Keywords:** alisol A 24-acetate, BMECs, microRNA-92a-3p, tight junctions, hypoxia

## Abstract

Blood brain barrier (BBB) dysfunction developed with aging is related to brain microvascular endothelial cells (BMECs) injury and losses of tight junctions (TJs). In the present study, we found that Alisol A 24-acetate (AA), a natural compound frequently used as treatment against vascular diseases was essential for BMECs injury and TJs degradation. Our experimental results showed that AA enhanced cell viability and increased zonula occludens-1 (ZO-1), claudin-5, and occludin expression in the oxygen-glucose deprivation (OGD)-induced BMECs. The exploration of the underlying mechanism revealed that AA restrained miR-92a-3p, a noncoding RNA involved in endothelial cells senescence and TJs impairment. To test the role of the miR-92a-3p in BMECs, the cells were transfected with miR-92a-3p mimics and inhibitor. The results showed that miR-92a-3p mimics inhibited cell viability and elevated lactate dehydrogenase (LDH) levels as well as suppressed ZO-1, claudin-5 and occludin expression, while the miR-92a-3p inhibitor reversed the above results. These findings were similar to the therapeutic effects of AA in the OGD-induced BMECs. Bioinformatics analysis and dual-luciferase assay confirmed ZO-1 and occludin were the target genes of miR-92a-3p mediated AA protective roles. In summary, the data demonstrated that AA protected against BMECs damage and TJs loss through the inhibition of miR-92a-3p expression. This provided evidence for AA application in aging-associated BBB protection.

## INTRODUCTION

Age-related vascular diseases are unresolved problems around the world. There is growing evidence that the microvascular endothelial cells (BMECs) of the brain play an important effect on age-related cerebral dysfunctions [1]. Dysfunction of the blood brain barrier (BBB) is observed as a critical step in the process of many central nervous system diseases, including aging and cognitive impairment [2–5]. It has been proposed that the aging process is accompanied by cerebral hypoperfusion, which is a microcirculatory disorder manifested by the alteration of endothelial cell functions and the promotion of the degradation of tight junctions (TJs) [6, 7]. BMECs form the highly selective BBB regulated by the endothelial tight junctions, which is involved in the preservation of the homeostasis of the cerebral tissue and normal cerebral blood flow. BMECs are interconnected by a continuous line of TJs that are crucial for BBB integrity [8]. Progressive dysfunction of BMECs and endothelial cell junctions during aging lead to BBB leakage, which is now considered as the most significant neuropathological change in the process of the age-related brain [9]. Cellular junctions among BMECs, including occludin, claudins, and zonula occludens (ZO), take part in the various paracellular signaling pathways [10–12]. They are usually downregulated by the hypoxic injury in age-related diseases, including aging, stroke, and dementia [1, 13–15]. It is thus important to consider the necessity of BBB permeability for delaying aging, and the importance of BMECs and TJs for BBB integrity that it is necessary to protect the BMECs and TJs to maintain BBB integrity for resisting aging [1, 16].

MicroRNAs (miRNAs) are a class of evolutionarily conserved, small noncoding RNAs that negatively regulate gene expression [17]. Several miRNAs have been shown to exert an important role in the BMECs and TJs with age-related cerebral vascular diseases [18, 19]. Among these miRNAs, miR-92a-3p is abundant in endothelial cells and participates in regulating vascular integrity and cell senescence [18–20]. Xia et al*.* indicated that miR-92a-3p promoted doxorubicin-induced cardiac senescence and miR-92a-3p inhibition repressed the senescence of cardiomyocytes [21]. Additionally, it is demonstrated that miR-92a-3p exerts a negative regulation on the expression of TJs-related genes [22, 23]. Based on the aforementioned research studies, we assumed that miR-92a-3p may participate in the TJs dysfunction in age-related vascular diseases.

Alisol A 24-acetate (AA) is extracted from alisma, a natural compound frequently used as treatment against vascular diseases. Previous studies have shown that AA repressed the release of inflammatory factors and oxidative stress [24, 25]. MiR-92a inhibitor improved the viability of endothelial cells through the reduction of oxidative stress [26]. Oxidative stress modified the expression of TJs and caused BBB disruption in rats [27]. In present experiments, we used an oxygen-glucose deprivation (OGD)-induced BMECs model to emulate the age-related cerebral hypoperfusion and investigate whether AA has a protective function in BMECs injury and TJs impairment caused by hypoxia-induced injury. The results showed that AA obviously enhanced cellular viability and increased TJs expression. Further, we found that AA repressed the miR-92a-3p upregulated and reversed the TJs degradation in the OGD-induced BMECs.

## RESULTS

### Cell viability after AA treatment

**Figure 1:**
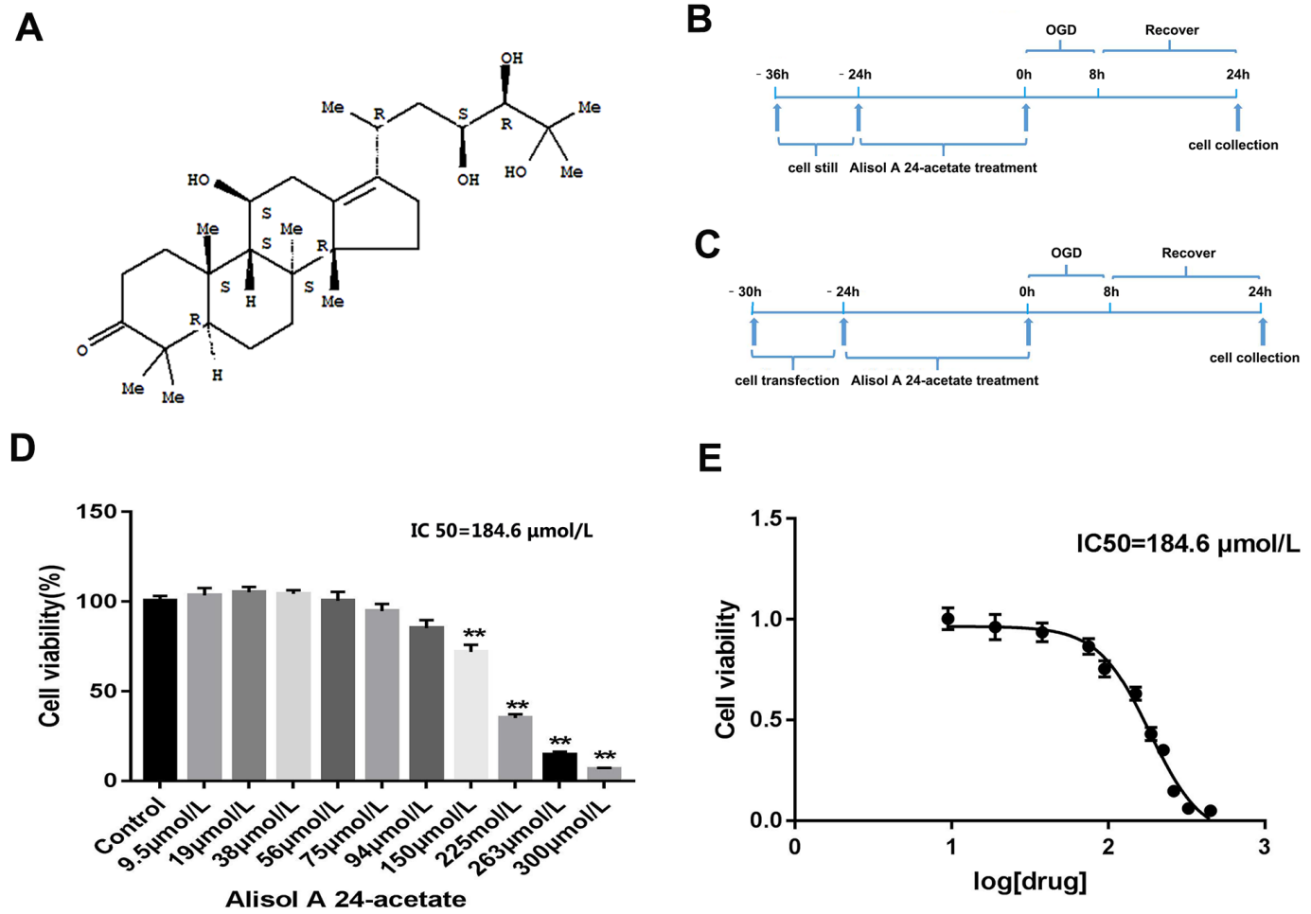
**Molecular structure of Alisol A 24-acetate, experiment design, and cell viability of Alisol A 24-acetate treatment**. (**A**) Molecular structure of Alisol A 24-acetate. (**B**) Schematic of experiment design of Alisol A 24-acetate treatment. (**C**) Schematic of experiment design of cell transfection and Alisol A 24-acetate treatment. (**D**) Cell viability was measured by the CCK-8 assay. (**E**) The IC50 was determined to be 184.6 μmol/L. Results are described as means ± standard deviations (SD) (n = 6) (**P < 0.01 vs. control group).

The molecular structure of AA is displayed in Figure 1A. The experimental processes are shown in Figure 1B, 1C. After AA treatment for 24 h, the viability of cells was examined. AA caused cell viability repression at doses above 150 μmol/L (*p* < 0.01, Figure 1D). The IC50 of AA was 184.6 μmol/L (Figure 1E). Hence, 1.9 μmol/L, 19 μmol/L, and 38 μmol/LAA were selected as the low (L), medium (M), and high (H) concentrations in the subsequent experiments.

### Roles of AA on the cells morphology and viability

**Figure 2:**
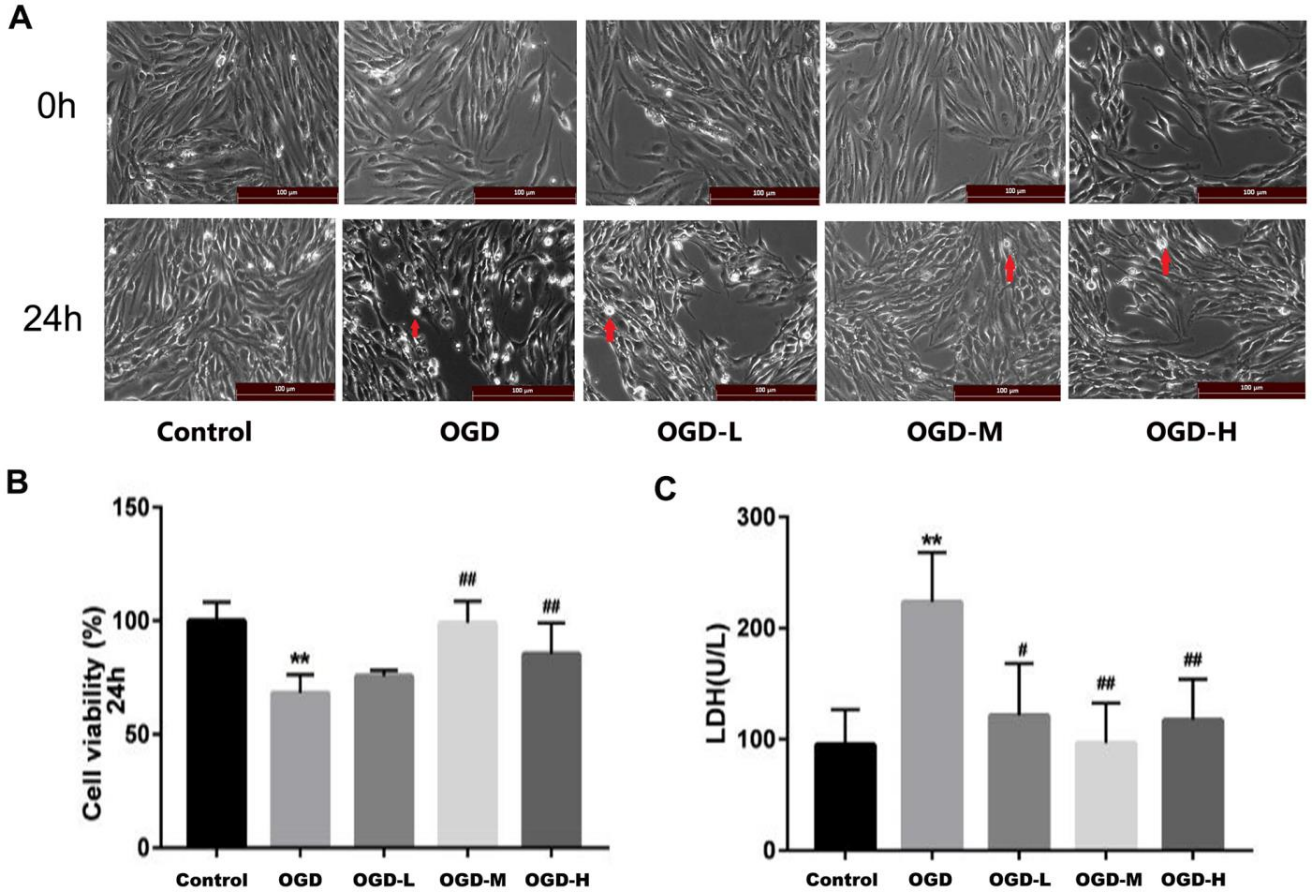
**Effects of Alisol A 24-acetate on the morphology and viability of bEnd.3 cells after OGD injury.** (**A**) Cells were observed under an inverted microscope after oxygen-glucose deprivation (OGD) (scale bar 100 μm). (**B**) Cell viability was calculated by the CCK-8 assay. (**C**) Secreted LDH levels in cell culture supernatant were determined by the LDH kit (OGD-L: 1.9 umol/L Alisol A 24-acetate; OGD-M: 19 umol/L Alisol A 24-acetate; OGD-H: 38 umol/L Alisol A 24-acetate). Results are described as means ± SD (n = 6) (**P < 0.01 vs. control group, ^##^*P* < 0.01 vs. OGD group).

The cells of each group were examined after OGD. In the control group, the cells displayed long spindle shape, closely arranged, and presented single-layer paving stone shape (Figure 2A). The growth of cells in the OGD group was suppressed, and many bright spots appeared in conjunction with necrotic morphological features. Treatment with AA promoted the states of cell growth. CCK-8 assays revealed that the OGD intervention decreased the cell activity compared with the control group (*p* < 0.01, Figure 2B). However, both the OGD-M and OGD-H groups had higher cell activities compared with the OGD group (*p* < 0.01, Figure 2B), thus suggesting that AA could improve cell viability after the OGD injury.

### Role of AA on lactate dehydrogenase (LDH) levels

LDH assay revealed that LDH release increased in the OGD group (*p* < 0.01, Figure 2C), whereas LDH spillage was dramatically reduced in the OGD-L group, OGD-M group, and OGD-H group in comparison to the OGD group (*p* < 0.05 or *p* < 0.01, Figure 2C), thus implying that AA (1.9 μmol/L to 38 μmol/L) could protect the cells against OGD injury.

### Roles of AA on the expression of TJs messenger RNA (mRNA)

**Figure 3:**
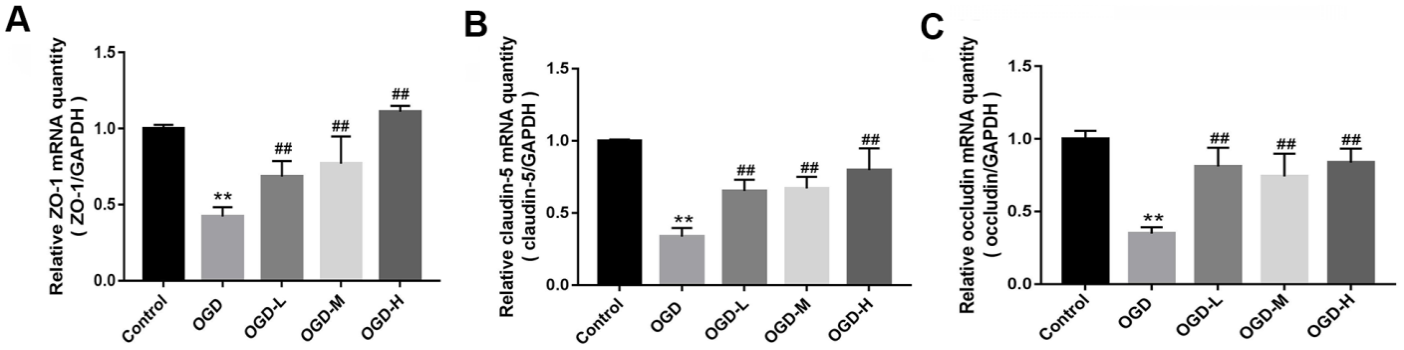
**Messenger ribonucleic acid (mRNA) expression of tight junctions (TJs) in OGD-induced bEnd.3 cells.** (**A**–**C**) Quantitative reverse transcription-polymerase chain reaction (qRT-PCR) was applied to detect the mRNA expression of zonula occludens (ZO-1), claudin-5, and occludin. The quantity was calculated. The housekeeping gene used to normalize the mRNA expression is GAPDH mRNA (OGD-L: 1.9 umol/L Alisol A 24-acetate; OGD-M: 19 umol/L Alisol A 24-acetate; OGD-H: 38 umol/L Alisol A 24-acetate). Results are described as means ± SD (n = 6) (**P < 0.01 vs. control group, ^##^*P* < 0.01 vs. OGD group).

Quantitative reverse transcription-polymerase chain reaction (qRT-PCR) indicated that TJs (ZO-1, claudin-5, and occludin) mRNA expressions of the OGD group were lower than those of the control group (*p* < 0.01, Figure 3A–3C). AA treatment (1.9 μmol/L, 19 μmol/L, and 38 μmol/L) raised the TJ mRNA expressions in the bEnd.3 cells (*p* < 0.01, Figure 3A–3C). Our results suggest that AA could improve the TJ mRNA expression in the OGD-induced cells.

### AA affects the expression of TJ proteins

**Figure 4:**
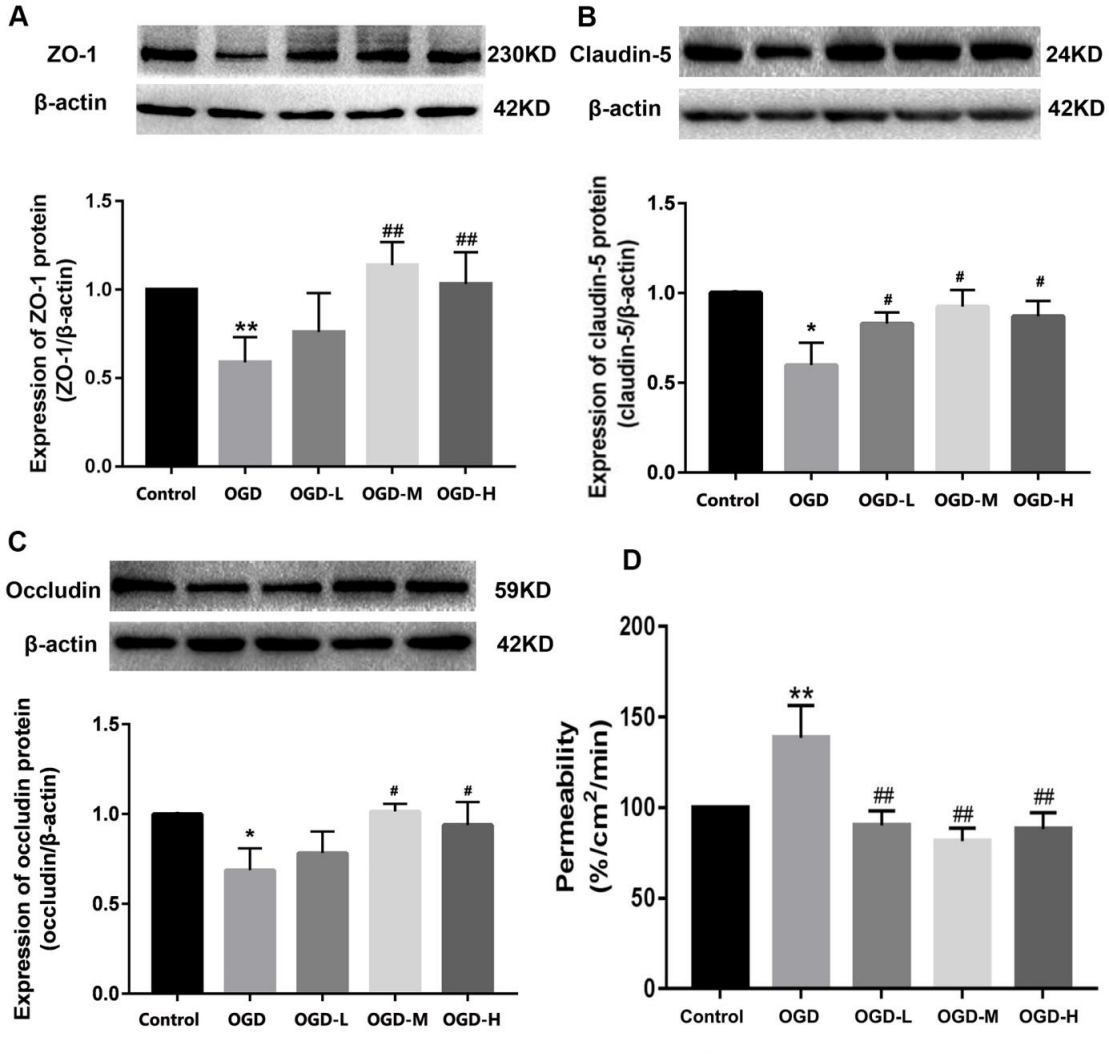
**Expression of TJ proteins and the permeability in OGD-induced bEnd.3 cells.** (**A**–**C**) Western blot analysis was performed to determine the expressions of ZO-1, claudin-5, and occludin. The amount of TJ proteins was quantified and normalized to that of β-actin. Results are described as means ± SD (n = 3). (**D**) Quantification of fluorescein isothiocyanate (FITC)-Dextran was conducted to assess cellular permeability. Results are described as the mean ± SD (n = 6) (OGD-L: 1.9 umol/L Alisol A 24-acetate; OGD-M: 19 umol/L Alisol A 24-acetate; OGD-H: 38 umol/L Alisol A 24-acetate) (*P < 0.05, **P < 0.01 vs control group; ^#^*P* < 0.05, ^##^*P* < 0.01 vs OGD group).

Western blot showed that the OGD group had the lower expression of ZO-1, claudin-5, and occludin compared with the control group. After intervention with AA, ZO-1, claudin-5, and occludin proteins were upregulated (*p* < 0.01 or *p* < 0.05, Figure 4A–4C). The results show that AA restores the downregulation of TJ proteins induced by OGD injury.

### AA attenuates the cell permeability

Quantification of the fluorescein isothiocyanate (FITC)-Dextran was applied to detect cellular permeability. The data displayed that OGD significantly increased cellular permeability, and AA treatment reduced the permeability in the OGD-induced bEnd.3 cells (*p* < 0.01, Figure 4D). It is thus implied that AA repressed the cells permeability through the elevation of the TJs expression.

### AA regulates the expressions of miR-92a-3p and miR-92a-1- 5p

**Figure 5:**
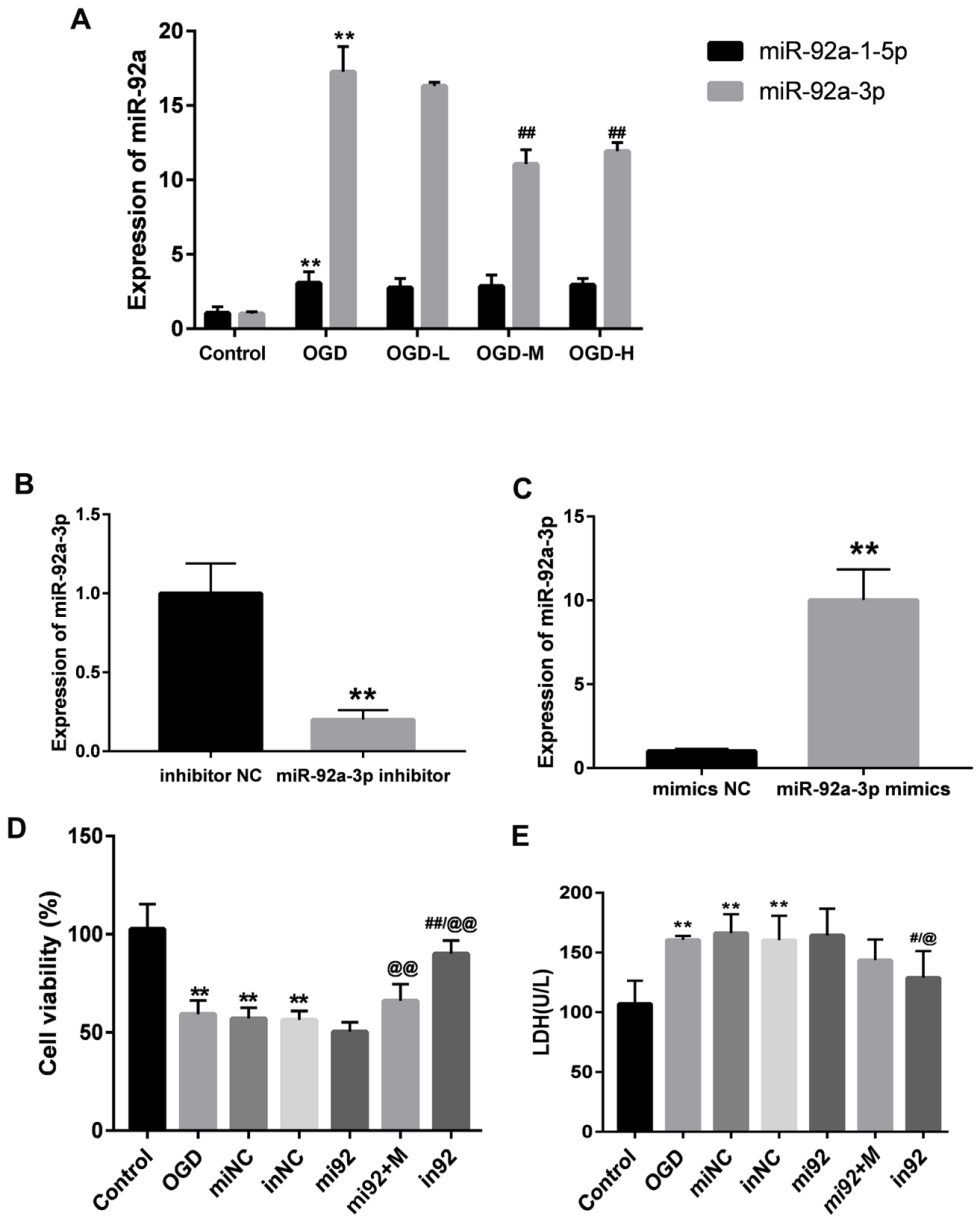
**Expressions of miR-92a-3p in OGD-induced bEnd.3 cells and transfection of miR-92a-3p mimics and inhibitor.** (**A**) Expressions of miR-92a-1-5p and miR-92a-3p in cells were detected by qPCR (miNC: mimics negative control; inNC: inhibitor negative control; mi92: miR-92a-3p mimics; mi92+M: miR-92a-3p mimics +19 umol/L Alisol A 24-acetate; in92: miR-92a-3p inhibitor). Results are presented as means ± SD (n = 6) (**P < 0.01 vs. control group; ^##^*P* < 0.01 vs. OGD group). (**B**, **C**) MiR-92a-3p expression of transfection with miR-92a-3p mimics and inhibitor assessed by qRT-PCR. (**D**) Cell viability of miR-92a-3p transfection was calculated. (**E**) LDH levels were calculated. Results are described as the mean ± SD (n = 6) (**P < 0.01 vs. control group; ^##^*P* < 0.01 vs. OGD group; ^@@^*P* < 0.01 vs. mi92a group).

The expressions of miR-92a-1-5p and miR-92a-3p were significantly enhanced after OGD (*p* < 0.01, Figure 5A). AA intervention decreased the miR-92a-3p expression after OGD injury (*p* < 0.01, Figure 5A), but there were no differences between the miR-92a-1-5p expression after the AA treatment. It is indicated that AA may specifically suppress miR-92a-3p upregulation in bEnd.3 cells.

### MiR-92a-3p mimics and inhibitor transfection affect bEnd.3 cell viability and LDH levels

We transfected the cells with 50 nM miR-92a-3p mimics and 50 nM miR-92a-3p inhibitor to investigate the function of miR-92a-3p on the OGD-induced cells. The expressions of miR-92a-3p between mimics/inhibitor and NC groups were different (*p* < 0.01, Figure 5B, 5C). This implied that mimics caused the upregulation of miR-92a-3p, and the inhibitor could repress the expression of miR-92a-3p. We then examined the effect of miR-92a-3p mimics and inhibitor on cell viability with the CCK8 assay. The cell viability of the OGD + mimics NC (miNC) and OGD + inhibitor NC (inNC) groups was similar to that of the OGD group (*p* < 0.01, Figure 5D), thus suggesting that transfection did not influence the effects on the cells caused by OGD injury. Compared with the control group, the cell viabilities of the OGD, miNC, and inNC groups was reduced (*p* < 0.01, Figure 5D), and the cell viability of the in92 group was higher than that of the mi92 and OGD groups (*p* < 0.01, Figure 5D). To investigate the effects of miR-92a-3p on LDH release, miR-92a-3p mimics and inhibitor were transfected into bEnd.3 cells. Compared with the control group, the LDH overflow in the OGD, miNC, and inNC groups increased significantly (*p* < 0.01, Figure 5E). The in92 group yielded a lower LDH release compared with the mi92 group (*p* < 0.01, Figure 5E). This revealed that the miR-92a-3p inhibitor mitigated the OGD injury and reversed the roles of miR-92a-3p mimics.

### Effect of miR-92a-3p mimics and inhibitor on the TJ proteins expression

**Figure 6:**
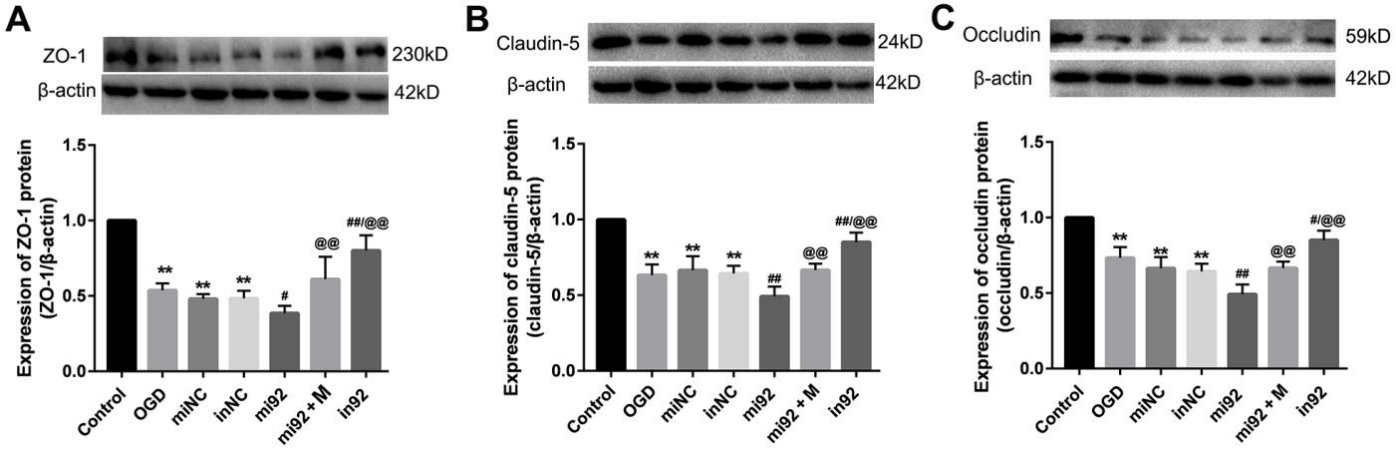
**Effects of miR-92a-3p transfection on the expression of TJ proteins.** (**A**–**C**) Western blot analysis was performed to determine the expression of ZO-1, claudin-5, and occludin. The amount of TJ protein was quantified and normalized to that of β-actin (miNC: mimics negative control; inNC: inhibitor negative control; mi92: miR-92a-3p mimics; mi92+M: miR-92a-3p mimics +19 umol/L Alisol A 24-acetate; in92: miR-92a-3p inhibitor). Results are described as means ± SD (n = 3) (**P < 0.01 vs. control group; ^#^*P* < 0.05, ^##^*P* < 0.01 vs. OGD group; ^@@^*P* < 0.01 vs. mi92a group).

Western blot outcomes showed that the expression of TJ proteins (ZO-1, claudin-5, and occludin) in the OGD, miNC, and inNC groups were lower than that of the control group (*p* < 0.01, Figure 6). The upregulation of miR-92a-3p in the mi92 group promoted the downregulation of TJ proteins compared with the OGD and miNC groups (*p* < 0.05, Figure 6A–6C). By contrast, the miR-92a-3p inhibitor reversed the downregulation of TJ proteins in the cells (*p* < 0.01, Figure 6A–6C). The expression of TJs (ZO-1, claudin-5, and occludin) in the in92a group increased significantly compared with the mi92 and OGD groups (*p* < 0.01, Figure 6A–6C). These data inferred that miR-92a-3p were closely associated with TJ proteins of the BMECs, while the miR-92a-3p inhibitor protected TJs against OGD injury. The expressions of TJ proteins increased significantly in the mi92 + M (miR-92a-3p mimics + 19 μmol/L AA) group compared with the mi92 group (*p* < 0.01, Figure 6A–6C), which indicated that AA could relieve the TJs degradation induced by miR-92a-3p overexpression in the BMECs.

### MiR-92a-3p inhibits the ZO-1 and occludin expression

**Figure 7:**
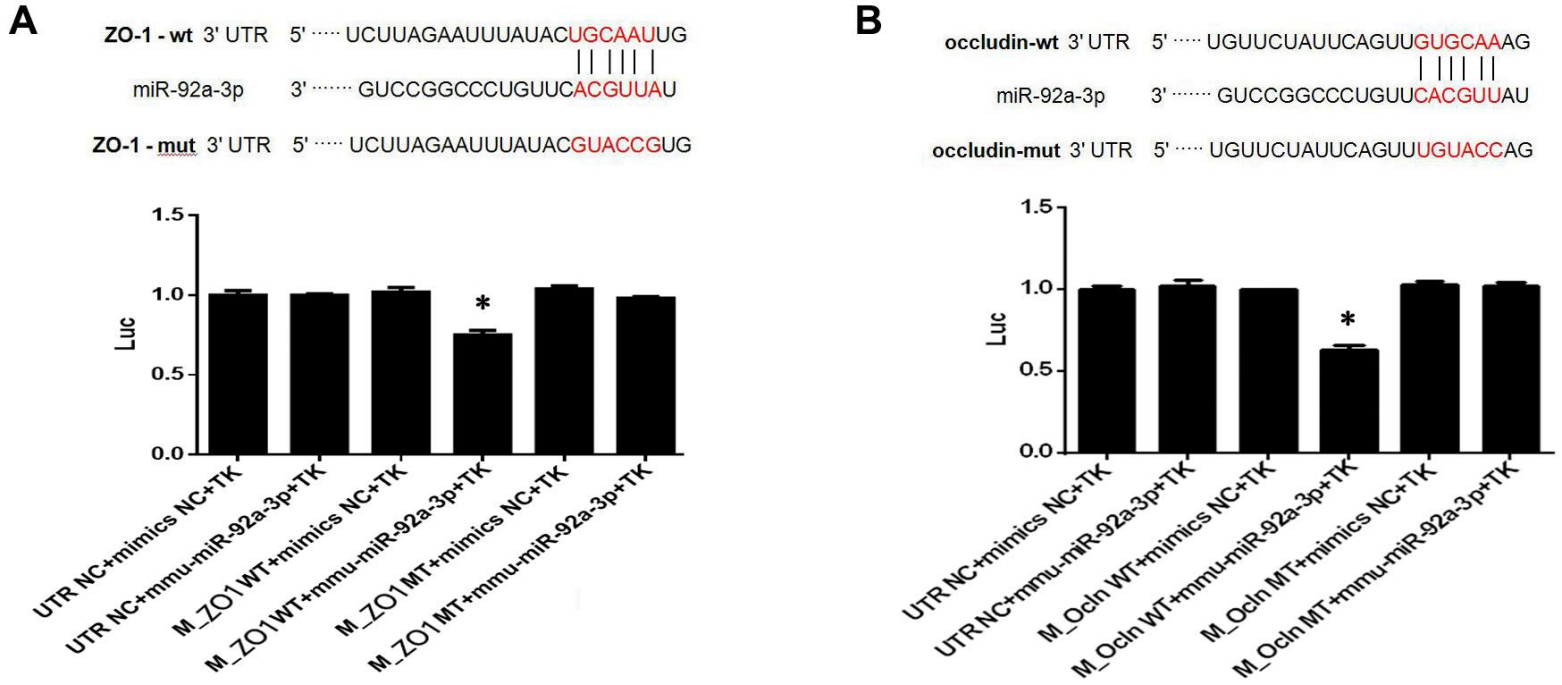
**MiR-92a-3p directly targets ZO-1 and occludin genes.** (**A**) MiR-92a-3p directly binds to ZO-1. Different types of luciferase reporter vectors were constructed: wild-type ZO-1 3’UTR (ZO-1 wild-type (WT)) and mutant-type ZO-1 3’UTR (ZO-1 MT). The above vectors were co-transfected into HEK-293 cells with miR-92a-3p mimics or mimics NC and examined for luciferase activity. (**B**) MiR-92a-3p directly binds to occludin. Different types of luciferase reporter vectors were constructed: wild-type occludin 3’UTR (Ocln WT) and mutant-type occludin 3’UTR (Ocln MT). The above vectors were co-transfected into HEK-293 cells with miR-92a-3p mimics or mimics NC and examined for luciferase activity. Each bar represents the mean ± SD of three independent experiments (*P < 0.05).

To investigate the function of miR-92a-3p on the expression of ZO-1 and occludin, Renilla luciferase reporter vectors, including the wild-type (WT) 3'-UTR of ZO-1 and occludin, mutants, and NC controls, were transfected into HEK-293 cells. The data showed that the luciferase activity of 3'-UTR WT ZO-1 and occludin transfection decreased compared with the control groups (*p* < 0.01, Figure 7). There was no difference of luciferase activity in cells which contained mutant ZO-1 and mutant occludin (Figure 7). Thus, the data demonstrated that the binding sites of miR-92a-3p in the ZO-1 and occludin, and the 3'-UTR sequence was crucial for the function of miR-92a-3p. Altogether, miR-92a-3p binds to the 3'-UTR sequences of ZO-1 and occludin, thus suggesting that miR-92a-3p negatively regulates the ZO-1 and occludin genes.

## DISCUSSION

BBB injury is one of the important pathophysiological mechanisms of aging-related diseases [1, 13]. BMECs exert a key effect on the preservation of the integrity of BBB and normal cerebral blood flow. BMECs have complex TJs, which can prevent the endogenous and exogenous substances from passing through the BBB [15]. Endothelial dysfunction and the breakdown of TJ proteins are the main changes in cerebral vascular diseases accompanied by aging [1, 18]. In this regard, the intervention of TJs in BMECs is an important treatment for age-related vascular diseases.

AA is one of the active triterpenoids extracts of alisma, and triterpenoids are known as the ingredients responsible for anti-inflammation [28]. Recently, it has been shown that Alisol 24-acetate inhibits the expression of inflammatory factors and reactive oxygen species through the mTOR pathway, thus suggesting that AA could exert a critical protection on endothelial cells of age-related vascular diseases [24, 25, 29].

There is growing evidence that the disruption of TJs (claudin-5, occludin, and ZO-1) can lead to functional changes of TJs [30–32]. The results showed that OGD induced a significant decrease in TJs expression, which was strongly associated with the upregulation of miR-92a-3p. It was estimated that miR-92a-3p accelerated cell senescence and suppressed TJs expression, while AA treatment led to a dramatical decrease of miR-92a-3p and increases of ZO-1, claudin-5, and occludin expression. LDH is known to be involved in the impairment of TJs [33]. When cells are injured by hypoxia, the LDH overflow and the cell permeability increase. In addition, the TJ proteins expression decreased [33, 34] in a similar manner to our results. In the present study, AA repressed the LDH release and reduced the cell permeability in the OGD injury cells. The results demonstrated that AA protected BMECs following the reduction of the LDH overflow and cell permeability, or the increase of the expression of TJs.

To investigate in-depth the underlying mechanism responsible for the function of AA, we examined whether the miRNA expressions in the OGD-induced cells were affected by AA. It is well known that miRNAs have emerged as important regulators of TJs proteins to modulate BBB function [35]. For example, miR-212 regulated the function of hypoxic BBB by targeting the transcription of TJs and TJs-associated proteins [36]. Inhibition of miR-155 obviously led to more pronounced reductions of the OGD-induced TJ proteins expression [37]. MiR-92a-3p was linked with endothelial injury, BBB impairment, and white matter damage [18]. The levels of miR-92a-3p expressions were upregulated in brain tissues and plasma during cerebral ischemia [38]. Our data showed that OGD stimulated miR-92a-3p and miR-92a-1-5p expressions in the cells. AA treatment specifically repressed the expression of miR-92a-3p, but did not affect the miR-92a-1-5p expression in OGD-induced BMECs. It was demonstrated that miR-92a-3p targeted the claudin-11 gene, which was closely related to intercellular TJs [22]. To test the function of miR-92a-3p on TJs, the mimics and inhibitor were transfected into bEnd.3 cells. We found that miR-92a-3p mimics inhibited cell viability and promoted LDH overflow as well as aggravated the degradation of TJs, all of which were reversed by the inhibitor. This indicates that miR-92a-3p is involved in the injury of BMECs. Further, we also found that the miR-92a-3p inhibitor alleviated the TJs degradation, which was similar to the therapeutic effect of AA in the OGD-induced BMECs. These results showed that miR-92a-3p was closely associated with the ZO-1, claudin-5, and occludin expressions, and miR-92a-3p could repress the expression of TJs (ZO-1, claudin-5, and occludin) directly or indirectly.

It has been demonstrated that the target genes of miR-92-3p contained TJ-related genes, such as claudin-11 [22]. No prior publication has reported that miR-92a-3p directly acts on ZO-1, claudin-5, and occludin. There were no claudin-5 3'-UTR binding sites sequences of miR-92a-3p in the Targetscan7.2 and miRDB databases. Thus, we cloned the 3^'^-UTR of the ZO-1 and occludin mRNA fragment and the mutants fragment which contained miR-92a-3p binding sites sequences and executed co-transfection of the luciferase reporter. We confirmed that miR-92a-3p decreased the ZO-1 and occludin expressions following its binding to the 3'-UTR sequences of ZO-1 and occludin mRNA. The expression of claudin-5 was affected by miR-92a-3p indirectly, or via the interactions of dysfunctional TJ. This suggests that the targeting TJs of miR-92a-3p are the important players in the OGD-induced BMECs and the miR-92a-3p inhibition is a promising treatment for the BMECs protection.

The ZO-1, claudin-5, and occludin expressions in the mi92a-M and in92a groups were higher than those of the mi92a group. MiR-92a-3p mimics partially weakened the protection of AA on OGD-induced BMECs. The inhibition of miR-92a-3p illustrated the protective effect against OGD injury as well as AA. The data further imply that AA can protect BMECs through the regulation of ZO-1, claudin-5, and occludin genes expression directly or indirectly. It is demonstrated that miR-92a-3p participates in the vascular cells senescence and TJs impairment [18, 19, 21]. AA could be used as a therapy against the progression of age-related vascular disease through the inhibition of miR-92a-3p expression.

In summary, miR-92a-3p is involved in the TJs degradation, and miR-92a-3p inhibition dramatically attenuates the TJs impairment. Treatment with AA specifically inhibits the miR-92a-3p upregulation and alleviates TJs degradation in BMECs. First, we verified that AA plays an important protective function in BMECs by repressing miR-92a-3p via targeting ZO-1 and occludin genes expression. This provides new evidence for AA application in BMECs protection.

## MATERIALS AND METHODS

### Cell culture

Murine brain microvascular endothelial cells (bEnd.3, Shanghai Jining Shiye Co. Ltd.) were cultured in high-glucose Dulbecco’s modified eagle’s medium (H-DMEM, Gibco, USA) with 10% fetal bovine serum (FBS, Gibco, USA) in 95% air and 5% CO_2_ at 37° C. The culture medium was replaced once every 2–3 days.

### Cell viability for selected action concentration of AA treatment

Alisol A 24-acetate (AA) (standard material, purity >98%) was purchased from Shanghai Yuanye Bio-Technology Co. Ltd (Shanghai, China). AA was dissolved in 50 μL dimethylsulfoxide (DMSO) in the form of a 20 g/L liquor. The liquor was added to serum-free medium for dilution, and the DMSO in the medium was no more than 0.1%. The latter was stored in the refrigerator at 4° C for subsequent use. A cell density of 5 × 10^3^ cells per well was cultured in 96-well plates. After 24 h, the medium was replaced with AA at different concentrations (0, 9.5, 19, 38, 56, 75, 94, 150, 225, 263, 300 μmol/L). After treatment for 24 h, 10 μL/well CCK8 solution (Boster, Wuhan, China) was added to the medium for an additional 1 h. The value was examined at an absorbance of 450 nm. The IC50 value was calculated with the software Graphpad Prism (version 7.0, GraphPad Software Inc., San Diego, CA, USA).

### OGD

When 80%–90% confluence was reached, the cells were washed with PBS (Gibco, USA) twice and then arranged to 5 groups: control, OGD, OGD-L, OGD-M, OGD-H. The OGD was set according to the protocol of Zuo et al. [39]. The cells in the OGD-L group, OGD-M group, OGD-H group were pretreated with 1.9 μmol/L, 19 μmol/L, and 38 μmol/L Alisol A 24-acetate for 24 h, followed by stimulation with OGD for 8 h, respectively.

### LDH assay

After the intervention, the LDH assay kit (Jiancheng Bio-engineering Institute, Nanjing, China) was used to detect the LDH levels in the supernatant of bEnd.3 cells according to the standardized instruction.

### qRT-PCR of TJ genes

The mRNA expressions of the bEnd.3 cells were quantified with the use of qRT-PCR according to standardized procedures. Briefly, the total RNA was extracted, and the pure RNA was reversed into cDNA using HiScript®QRT SuperMix for qPCR (Vazyme, Nanjing, China) following the manufacturer's protocol. The cDNA underwent PCR with the use of the ABI 7500 real-time PCR system (Applied Biosystems, Foster City, CA, USA). Specifically, qRT-PCR was performed with ChamQ SYBR qPCR Master Mix (Vazyme, Nanjing, China). The expression of mRNA was quantified subject to the following conditions: initial denaturation at 95° C for 30 s, denaturation at 95° C for 10 s, annealing at 60° C for 30 s, 40 cycles of extension at 60° C for 30 s, and a final set of cycles of extension at 60° C for 20 min. The primer sequences were as follows: ZO-1 F: 5′-CATAGTTCAACA CAGCCTCCAG and R: 5′- CCATCCTCATCTTCATCTTCTTCC-3′, claudin-5 F: 5′-TGGCACTCTTTGTTACCTTGAC-3′ and R: 5′-GCACCGTCGGATCATAGAA C-3′, occludin F: 5′-ATGGCTGCTGCTGATGAATA-3′ and R: 5′-CTTGATGTGC GATAATTTGCTCTT-3′. GAPDH F: 5′-TGGAAAGCTGTGGCGTGATG-3′ and R 5′-TACTTGGCAGGTTTCTCCAGG-3′. The levels of TJs (ZO-1, claudin-5, and occludin) genes were normalized to GAPDH.

### Western blot analysis

The cells were digested by trypsin, evenly absorbed, and centrifuged. The protein was extracted with RIPA buffer. The protein was boiled for 10 min with 6× loading buffer (5:1, v/v). The protein was then separated by 10% polyacrylamide gel electrophoresis and transferred to a PVDF membrane. The membrane was blocked in 5% bovine serum albumin at room temperature for 2 h, incubated with rabbit anti-mouse claudin-5 (24 kDa, 1:1000, Abcam, Cambridge, England, UK), occludin (59 kDa, 1:1000, Abcam, Cambridge, England, UK), ZO-1 (230 kDa, 1:1000, proteintech, Proteintech Group, Rosemont, IL, USA), and β-actin (42 kDa, 1: 8000, proteintech, Proteintech Group, Rosemont, IL, USA) primary antibodies at 4° C overnight, respectively. The blot was incubated with HRP-conjugated goat anti-rabbit or goat anti-mouse secondary antibody (1:5000, proteintech, Proteintech Group, Rosemont, IL, USA) at room temperature for 2 h. The blots were developed with an ECL kit (Boster, China). The grayscale values of the bands were measured using Image-Lab (Bio-Rad, Hercules, CA, USA). These experiments were repeated three times. The average value of the control group was calculated, and the ratio of all target proteins to β-actin was compared with the average value of the control group.

### Endothelial permeability assay

The endothelial permeability assay was performed with FITC-Dextran (70 kDa) extravasation for quantification across bEnd.3 cells seeded in transwell chambers. Briefly, 2 × 10^4^ cells per well were incubated on pore polycarbonate membrane (0.4 μm) inserts in 6.5 mm transwell plates (Corning, NY, USA) and grown for 5 days to confluence. Cells were treated with or without OGD/R and AA as described previously. After OGD exposure, the medium in the upper compartment was replaced with medium which contained 20 μg/mL FITC-Dextran (70 kDa, XiaoYou, Hangzhou, China) and PBS was added in the lower compartment. After culturing for 1 h in the dark, the relative fluorescence was measured that crossed the lower chamber using Thermo Fisher Multiskan FC (Thermo Fisher Scientific, Waltham, MA, USA) at wavelengths of 493 nm for excitation and 518 nm for emission.

### qPCR for microRNAs

Total RNA was extracted from bEnd.3 cells with the use of a RNAmisi microRNA rapid extraction kit (Aidlab Biotechnologies Co, Ltd.), and transcribed reversely into cDNA. The expressions of miR-92a-3p and miR-92a-1-5p were measured using SYBR® Premix Ex Taq™ II (Takara) and ABI 7500 Real-Time PCR system (Applied Biosystems, Foster City, CA, USA) based on the following conditions: denaturation at 95° C for 10 s, annealing at 60° C for 30 s, 30 cycles of extension at 72° C for 45 s, and a final set of cycles of extension at 72° C for 7 min. U6 served as an internal reference. According to GenBank, the primers were designed as follows: miR-92a-3p, F: 5′-CCGCGTAT TGCACTTGTCCC-3′ and R:5′-AGTGCAGGGTCCGAGGTATT-3′; miR-92a-1-5p F: 5′-CCGAGGTTGGGATTTGTCGC-3′ and R: 5′-AGTGCAGGGT CCGAGGTATT-3′; U6, F: 5′-CTCGCTTCGGCAGCACATATACT-3′ and R: 5′-AC GCTTCACGAATTTGCGTGTC-3′. The levels of miRNA were calculated with the 2−ΔΔC t (ΔΔCt = (Ct miRNA1 - Ct U6) - (Ct miRNA2 - Ct U6)) relative quantification method. U6 was used as the internal control.

### Cell transfection

MiR-92a-3p mimics, inhibitor, mimics NC, and inhibitor NC were obtained from GenePharma (Shanghai, China). The miR-92a-3p mimics sequences were: 5′-UAUUGCACUUGUCCCGGCCU-3′ and the corresponding negative control (mimics NC) sequences: 5′-UUCUCCGAACGUGUCACGUTT-3′. The miR-92a-3p inhibitor sequences: 5′-CAGGCCGGGACAAGUGCAAUA-3′ and the corresponding negative control (inhibitor NC) sequences: 5′-CAGUACUUUUGUGUAGUACAA-3′. The GP-transfect-Mate (GenePharma, Shanghai, China) was used to transfect cells. The cells were incubated at 1 × 10^5^ cells/well in six-well cell culture plates, and were grown to 60%–70% confluence. The miR-92a-3p mimics (mi92, 50 nM), inhibitor (in92, 50 nM), inhibitor NC (inNC, 50 nM) and mimics NC (miNC, 50 nM) were added directly to the cells. The cells were assigned randomly to 7 groups: control, OGD, miNC, inNC, mi92, mi92 + M, in92. The miNC, inNC, mi92, and in92 groups were transfected respectively. The mi92 + M group was transfected respectively for 6 h, and then intervention with 19 μmol/L AA for 24 h. The group details and process are displayed in Figure 1C. After transfection, the OGD, miNC, inNC, mi92, mi92 + M, and in92 groups were followed by stimulation with OGD, respectively. Cell viability and expressions of TJ proteins were quantified.

### Validation of predictive target genes of miR-92a-3p

To verify whether ZO-1 and occludin were targets of miR-92a-3p, Renilla luciferase control reporter vectors were inserted with WT and mutant 3′-UTR of ZO-1 and occludin genes (pRL-TK, Promega, Madison, Wisconsin, USA) respectively. The miR-92a-3p mimics or mimic NC and pRL-TK vectors were co-transfected into HEK-293 cells with lipofectamine®2000 (Invitrogen, Thermo, Waltham, MA, USA). Luciferase activity was detected by dual-luciferase assays (Genomeditech, Shanghai, China), and normalized to a Renilla luciferase expression.

### Statistics

All experimental results are reported as mean ± standard deviation. The statistical calculations were conducted with the use of SPSS (version 21.0, SPSS Inc., Chicago, IL, USA). The data were assessed by one-way analysis of variance and then by Fisher’s protected least-significant difference test or Student’s t-test to compare two interventions. *P* values < 0.05 were considered statistically significant.
